# A new risk factor indicator for papillary thyroid cancer based on immune infiltration

**DOI:** 10.1038/s41419-020-03294-z

**Published:** 2021-01-06

**Authors:** Zhou Yang, Xiyi Wei, Yitong Pan, Jingyuan Xu, Yan Si, Zhijun Min, Bo Yu

**Affiliations:** 1 Department of General Surgery, Shanghai Key Laboratory of Vascular Lesions Regulation and Remodeling, Shanghai Pudong Hospital, Fudan University Pudong Medical Center, 201399 Shanghai, China; 2 First Clinical Medical College of Nanjing Medical University, 210009 Nanjing, Jiangsu China; 3 Department of Bioinformatics, School of Biomedical Engineering and Informatics, Nanjing Medical University, 211116 Nanjing, Jiangsu China; 4 Department of Gastroenterology, The Second Affiliated Hospital of Xi’an Jiaotong University, 710004 Xi’an, China; 5 Department of Vascular Surgery, Huashan Hospital, Fudan University, 200040 Shanghai, China

**Keywords:** Cancer microenvironment, Tumour immunology

## Abstract

Increasing evidence has indicated a close association between immune infiltration in cancer and clinical outcomes. However, related research in thyroid cancer is still deficient. Our research comprehensively investigated the immune infiltration of thyroid cancer. Data derived from TCGA and GEO databases were analyzed by the CIBERSORT, ESTIMATE, and EPIC algorithms. The CIBERSORT algorithm calculates the proportions of 22 types of immune cells. ESTIMATE algorithm calculates a stromal score to represent all stromal cells in cancer. The EPIC algorithm calculates the proportions of cancer-associated fibroblasts (CAFs) and endothelial cells (ECs), which are the main components of stromal cells. We analyzed the correlation of immune infiltration with clinical characteristics and outcomes of patients. We determined that the infiltration of CD8^+^ T cells improved the survival of thyroid cancer patients. Overexpression of immune checkpoints was closely related to the development of thyroid cancer. In general, stromal cells were associated with the progression of thyroid cancer. Interestingly, CAFs and ECs had opposite roles in this process. In addition, the BRAF^V600E^ mutation was related to the upregulation of immune checkpoints and CAFs and the downregulation of CD8^+^ T cells and ECs. Finally, we constructed an immune risk score model to predict the prognosis and development of thyroid cancer. Our research demonstrated a comprehensive panorama of immune infiltration in thyroid cancer, which may provide potential value for immunotherapy.

## Introduction

In recent years, the incidence of thyroid cancer has been increasing worldwide. According to the tissue of origin and morphology, thyroid cancer can be divided into papillary thyroid cancer (PTC), follicular thyroid cancer (FTC), medullary thyroid cancer (MTC), poorly differentiated thyroid cancer (PDTC), and anaplastic thyroid cancer (ATC). Among them, PTC is the most common type, accounting for 60% of all pathological types. In addition, PTC and FTC are both types of differentiated thyroid cancer (DTC), which has a benign prognosis^1–3^. In contrast, the prognosis of ATC is very poor, and the median survival time of patients is only 7–10 months^4^. The prognosis of PDTC is between that of DTC and ATC^5^. In the past 10 years, the overall survival rate of patients with thyroid cancer of nearly 10% has not been significantly improved. Therefore, searching for new targets and therapies is required^6^.

Tumor cells combined with their microenvironment function as a whole unit, which is also called the tumor microenvironment (TME). The TME is mainly composed of tumor cells, immune cells, stromal cells, microvessels, various cytokines, and chemokines. All components in the TME have important roles in tumor initiation and progression. Among them, immune cells are the most crucial cluster that may affect the clinical outcomes of thyroid cancer^7^. It has been reported that the composition and function of tumor-infiltrating immune cells vary with the host immune status and have latent prognostic value. Cancer-associated fibroblasts (CAFs), which are included in the stromal cells, are also an important component of the TME. By secreting a variety of growth factors, chemokines, and proteases, CAFs regulate the recruitment and function of immune cells^8^. In addition, tertiary lymphoid structures (TLSs) exist, which include clusters of immune cells around tumor tissue where T cell and B-cell responses occur. TLSs have been reported in various types of cancer and are associated with prognosis^9^.

Notably, the TME could be effectively targeted by immunotherapy and associated with the clinical outcomes of patients^10^. Tumor immunotherapy is a treatment that eliminates tumor cells by restoring and maintaining normal antitumor immune responses and includes immune checkpoint inhibitors, therapeutic antibodies, cell therapy, and small molecule inhibitors. In particular, immune checkpoint inhibitors (ICIs) have changed the therapeutic landscape of advanced malignancies^11^. In thyroid cancer and other thyroid diseases, several studies have also demonstrated the potential value of ICIs^12,13^.

Several studies have revealed immune cell infiltration and immune checkpoint expression in thyroid cancer. Many studies have confirmed the high expression of CD4^+^ T cells, CD8^+^ T cells, and CD69 in patients with thyroid cancer such as MTC, which indicates obvious T cell reaction in thyroid cancer patients. High expression of PDL1, the main ligand of PD1, was also found in DTC, ATC, and MTC subtypes. Additionally, Joyce JA found that dendritic cells, rarely found in normal thyroid tissues, increased in thyroid cancer tissues, thus inhibiting the immune response^14^. Myeloid-derived suppressor cells (MDSCs), Neutrophils, NK Cells, Mast Cells (MCs) were also confirmed to interact with thyroid tumor cells through chemokines, adipokines, and cytokines^7^. However, these studies have been basically limited to a single pathological type, single immune cell, or single immune checkpoint^15,16^. More research focusing on multiple aspects of the TME needs to be carried out. Our research investigated 22 types of immune cells, various immune checkpoints, stromal cells including CAFs and endothelial cells (ECs), and TLSs in thyroid cancer based on data from The Cancer Genome Atlas (TCGA) database and the GEO database. The correlation between immune infiltration and clinical characteristics, including survival, pathological types, pathological stages, and gene mutation, was also analyzed. Our research may provide potential value for the immunotherapy of thyroid cancer.

## Material and methods

### Data acquisition

In all analyses of PTC, the data were derived from the TCGA database (the TCGA database contains only PTC data). We identified and downloaded the transcriptome data from the TCGA database through the R package “TCGA-Assembler”, including 58 cases of healthy thyroid tissues and 510 cases of PTC. Additionally, relevant clinical characteristics were also obtained and are shown in Table S1. In the analysis of pathological types, the data were derived from the GEO database. Four datasets based on the same RNA-sequencing platform, GPL-570, were merged, GSE33630 (49 cases of PTC and 11 cases of ATC), GSE65144 (12 cases of ATC), GSE76039 (17 cases of PDTC and 20 cases of ATC), and GSE82208 (27 cases of FTC)^17–19^. In addition, 33 cases of radiation-induced PTC (Exposed to Chernobyl Radiation, ECR^+^) and 32 non-ECR (ECR^−^) cases of PTC were derived from GSE35570 and analyzed separately^20^. All data derived from the TCGA and GEO databases were normalized to gene expression data through the R software package “Limma”.

### Assessment of immune cells

CIBERSORT is a deconvolution algorithm using the expression values of 547 genes to characterize the composition of immune cells in tissues. In this study, we used this algorithm to estimate the relative proportion of 22 infiltrating immune cell types based on gene expression. We uploaded the normalized gene expression data to the CIBERSORT website (http://cibersort.stanford.edu/) and set the algorithm to 1000 rows. *P* < 0.05 was considered to be statistically significant^21^.

### Assessment of stromal cells

All stromal cells, including CAFs, ECs, mesenchymal stem cells (MCSs), and pericytes, are included in the stromal score provided by the ESTIMATE algorithm^22^. The proportions of CAFs and endothelial cells (ECs) were analyzed by the EPIC algorithm^23^. Similar to the assessment of immune cells, normalized gene expression data were uploaded to the EPIC website (https://gfellerlab.shinyapps.io/EPIC_1-1/) to acquire the final proportion based on the expression of a series of gene makers of CAFs (ADAM33, CLDN11, COL1A1, etc.) and ECs (CDH5, CLDN5, CLEC14A, etc.).

### Assessment of tertiary lymphoid structures

The assessment of TLSs was performed according to the methods in previous research^24^. The geometric mean of a series of chemokines (CCL2, CCL3, CCL4, CCL5, CCL8, CCL18, CCL19, CCL21, CXCL9, CXCL10, CXCL11, and CXCL13) related to TLSs was adopted as the TLS score. Cases with TLS scores greater than the third quartile were classified as TLS^+^, and cases with TLS scores less than the third quartile were classified as TLS^−^.

### Gene ontology, KEGG pathway, and Gene set enrichment analysis

Gene list was uploaded to Database for Annotation Visualization and Integrated Discovery (DAVID, david.ncifcrf.gov/) online tool for Gene Ontology (GO) and KEGG pathway analysis. Concreate pathways and *P*-values were got and visualized by R software. Gene set enrichment analysis (GSEA) was performed using gsea-3.0, downloaded from the GSEA database (http://software.broadinstitute.org/gsea/index.jsp) with the built-in standard datasets.

### Patients and specimens

Seventy-two PTC specimens were collected from July 2013 to July 2019. Patients with the following criteria were excluded from participation: had received adjuvant chemotherapy or radiotherapy prior to surgery; had additional cancer diagnoses. All patients were classified according to the 7th edition of the TNM staging system 23. Postoperative adjuvant therapies were performed, according to standard schedules and doses. All participating patients gave their written informed consent. This study was approved by the Ethical Committee of Shanghai Pudong Hospital. The clinical data of all CRC patients were shown in Supplementary Table S2.

### Immunohistochemical (IHC) staining

IHC was performed on paraffin-embedded sections. The sections were deparaffinized in xylene and hydrated with decreasing concentrations of ethanol (100, 90, 80, 75%) for 3 min each time and microwaved-heated in sodium citrate buffer for antigen retrieval. Then, the sections were blocked in 5% BSA and incubated with anti-CD8 rabbit polyclonal antibody (1:1000, Abcam, UK) at 4 °C overnight. Next, the sections were treated with horseradish peroxidase (HRP)‑conjugated rabbit secondary antibody (1:200; ProteinTech Group, Inc., Wuhan, China) for 60 min at room temperature; then, 3,3′‑diaminobenzidine development (DAB Substrate Chromogen System; Dako, Denmark) and hematoxylin staining were performed. The sections were fixed and images were obtained with an inverted microscope (Olympus IX71, Japan).

### Cell culture and co-culture

Human umbilical vein endothelial cells (HUVECs) were purchased from Allcells, Inc. (Alameda, CA, USA) and cultured in Endothelial Cell Medium (ECM; ScienCell Research Laboratories, Carlsbad, CA, USA) supplemented with 10% fetal bovine serum (FBS, Invitrogen, Carlsbad, CA, USA). Human thyroid fibroblasts were purchased from ScienCell Research Laboratories and cultured in Dulbecco’s modified Eagle’s medium (DMEM) supplemented with 10% FBS. TPC-1 cells (human PTC cell line) were obtained from the University of Colorado Cancer Center Cell Bank. All cells were cultured at 37 °C in a 5% CO_2_ atmosphere. All experiments were performed with mycoplasma-free cells.

For cell co-culture, 2 × 10^5^ TPC-1 were seeded in the upper chambers of a Transwell system (24-well insert, 4 μm pore size; BD Biosciences, Bedford, MA, USA), 10^5^ HUVECs or fibroblasts were cultured in the lower chambers. After 24 h, CCK8 assay was applied to measure the proliferation of HUVECs or fibroblasts.

### CD8^+^ T cells apoptosis assay

Peripheral blood mononuclear cells (PBMCs) from healthy human donors were isolated using Lymphoprep density gradient centrifugation (Accurate Chemical). CD8^+^ T cells were further purified from PBMCs by negative selection using the EasySep Human CD8^+^ T Cell Enrichment Kits (STEMCELL Technologies Inc.). Then, CD8^+^ T cells were activated by anti-CD3/CD28 Dynabeads (Thermo Fisher Scientific, Waltham, MA, USA) for 48 h. Activated CD8^+^ T cells were co-cultured with the TPC-1 cells at a 10:1 ratio for 24 h. Finally, CD8^+^ T cells were harvested and measured by flow cytometer using Annexin V-PE/7-AAD Apoptosis Detection Kit (BD Biosciences).

### Plasmid transfection

pcDNA3.1-BRAF^wt^, pcDNA3.1-BRAF^V600E^, and pcDNA3.1-TNNT1 plasmids were purchased from HedgehogBio, Inc. (Shanghai, China). For transfection, 10^6^ cells were seeded in a 6 cm dish and cultured at 37 °C. After 18 h, 2 μg plasmid accompanied with 10 μl Lipofectamine 3000 (Invitrogen, Inc.) were added in culture media. Subsequently, cells were further cultured at 37 °C for 36 h.

### Statistical analysis

All analyses were performed using SPSS 23.0 and R 3.5.3. All statistical tests were two-sided, and a *P*-value < 0.05 was considered statistically significant. Continuous variables that conformed to the normal distribution were compared with the use of an independent *t*-test for comparison between groups, while continuous variables with skewed distribution were compared with the Mann–Whitney *U* test. The correlation matrix was constructed by R software based on the Pearson correlation coefficient. The relationship between immune cell infiltration and overall survival was analyzed through the Kaplan–Meier method, which was evaluated by the log-rank test. Time-dependent ROC curves were used to analyze the sensitivity and specificity of the recurrence prediction model. The univariate regression model was used to analyze the effects of individual variables on survival, and the multivariate Cox regression model was used to confirm the independent impact factors associated with survival. The nomogram was constructed with regression coefficients based on the Cox analysis.

## Results

### Immune infiltration in papillary thyroid cancer is closely related to survival

**Fig. 1:**
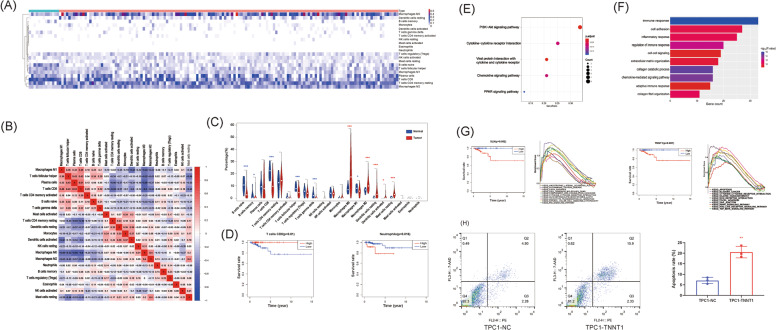
Immune infiltration in papillary thyroid cancer is closely related to survival. **A** Heatmap of 22 types of immune cells in papillary thyroid cancer and healthy thyroid tissues derived from the TCGA database. **B** The correlation matrix of 22 types of immune cells in papillary thyroid cancer (PTC). **C** Comparison of each type of immune cell between PTC and healthy thyroid tissues. **D** The correlation of immune cells and overall survival (OS). **E** KEGG pathway analysis of genes associated with downregulation of CD8^+^ T cells. **F** Gene Ontology (GO) analysis of genes associated with downregulation of CD8^+^ T cells. **G** Correlation of genes associated with downregulation of CD8^+^ T cells and overall survival (OS), including GSEA results. **H** The apoptosis rated of CD8^+^ T cells co-cultured with TPC1-TNNT1 (overexpression of TNNT1) and TPC1-NC (negative control). (**P < 0.01, The color for */# indicates the column with the same color was overexpressed).

The proportion of 22 types of immune cells in PTC and healthy thyroid tissues was calculated by the CIBERSORT algorithm based on data from the TCGA database (Fig. 1A). We also demonstrated a close negative or positive connection between each type of immune cell in PTC via a correlation matrix (Fig. 1B). Furthermore, we investigated the differences in immune cell proportions between PTC and healthy thyroid tissues. The proportions of naive B cells, memory B cells, CD8^+^ T cells, regulatory T cells (Tregs), gamma delta T cells (γδT cells), follicular helper T (TFH) cells, and M1 macrophages were significantly decreased. However, the proportions of M0 macrophages, M2 macrophages, resting dendritic cells (DCs), activated DCs, and resting mast cells were significantly increased (Fig. 1C). Subsequently, we investigated the relationship between each immune cell type and overall survival (OS) in PTC patients. We found that a low proportion of CD8^+^ T cells was associated with worse OS (*P*-value = 0.01), whereas a low proportion of neutrophils indicated better OS (*P*-value = 0.016, Fig. 1D). We also demonstrated the correlation of OS with CD8^+^ T cells and neutrophils in pan-cancer through the TIMER website tool (https://cistrome.shinyapps.io/timer/) based on TCGA database^25^. CD8^+^ T cells and neutrophils were closely related to OS in various cancers, including bladder urothelial carcinoma (BLCA), cholangiocarcinoma (CHOL), and so on. Interestingly, the same correlation is not always reported in other tumors, especially in uveal melanoma (UVM). Totally opposite from that in PTC, a low proportion of CD8^+^ T cells but a high proportion of neutrophils was associated with better OS in UVM (Supplementary Fig. S1). It seems the association of immune cells and survival was capricious in different tumors, which emphasized the uniqueness of immune infiltration in PTC. Furthermore, we extracted data on genes responsible for downregulation of CD8^+^ T cells and performed Gene Ontology (GO) and KEGG pathway analyses. Various pathways and annotations were identified as responsible for the downregulation of CD8^+^ T cells, including pathways involving cytokines, PI3K/Akt and PPAR, extracellular matrix, collagen catabolic processes, and so on (Fig. 1E, F). We further analyzed the association between genes responsible for the downregulation of CD8^+^ T cells and OS to identify key hub genes. First of all, we divided the patients into high and low-expressed groups according to the median expression of CD8^+^ T cells. In addition, we analyzed the differentially expressed genes between the high and low expression groups and selected the genes with low expression in the high CD8^+^ T cells group. Among these differentially expressed genes, only high expression of SLN and TNNT1 was associated with both downregulated CD8^+^ T cells and worse OS. GSEA was further performed to identify SLN- and TNN1-associated pathways. Immune-associated pathways (pathways involving cytokine receptors, NOD-like receptors, etc.) might be responsible for regulating CD8^+^ T cells, and SLN and TNN1 were also associated with various pathways responsible for the growth and invasion of tumors (apoptosis, p53, TGF-β, etc, Fig. 1G). Furthermore, we selected TNNT1 for verification, as it was closely related to apoptosis pathways. TNNT1 overexpressed cell line TPC1-TNNT1 and negative control TPC1-NC were constructed. CD8^+^ T cells were purified from PBMC and co-cultured with TPC-1 cells. As expected, TPC1-TNNT1 promoted the apoptosis rate of CD8^+^ T cells (Fig. 1H).

### Immune infiltration was associated with different clinical characteristics

**Fig. 2:**
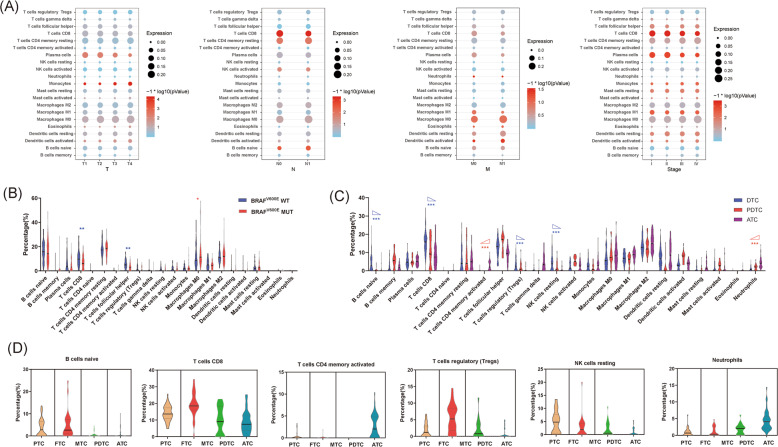
Immune infiltration is associated with different clinical characteristics. **A** Correlation of immune cells and different pathological stages (pathological TNM stage and pathological stage) of PTC. **B** Correlation of immune cells and BRAF^V600E^ mutation in PTC. **C** Correlation between immune cells and different pathological types of thyroid cancer. DTC (differentiated thyroid carcinoma, including PTC and FTC), PDTC (poorly differentiated thyroid carcinoma), ATC (anaplastic thyroid cancer). Red triangle: continual increase; clue triangle: continual decrease. **D** Correlation of immune cells and specific pathological types of thyroid cancer. (*P < 0.05, ***P < 0.001).

We further investigated the correlation of immune cells with different pathological stages of PTC (Fig. 2A, detailed statistical values were showed in Supplementary Table S3). As the pathological T stage advanced, monocytes, eosinophils, and activated DCs increased, whereas plasma cells decreased. As the pathological N stage advanced, naive B cells and CD4 memory resting T cells increased, whereas CD8^+^ T cells and activated NK cells decreased. With the development of the pathological M phase, the number of activated DCs and neutrophils increased. With the advancement of the pathological stage, monocytes, resting dendritic cells, activated dendritic cells, resting mast cells and activated mast cells increased, CD8^+^ T cells and plasma cells decreased, follicular helper T cells and macrophages M1 decreased first and then increased. As BRAF^V600E^ mutation is the most common gene mutation in PTC and is associated with poor prognosis, we further evaluated the correlation between immune cells and BRAF^V600E^ mutation. We demonstrated that in PTC with the BRAF^V600E^ mutation compared to PTC with wild-type BRAF, M0 macrophages were increased, whereas CD8^+^ T cells and TFH cells decreased (Fig. 2B). Subsequently, we further investigated the correlation between immune cells and different pathological types of thyroid cancer based on GEO data. All data were derived from five GEO data sets derived from the same sequencing platform. The pathological types of thyroid cancer were divided into DTC (PTC and FTC), PDTC, and ATC, with a progressively decreasing prognosis. We demonstrated activated CD4^+^ T cells and neutrophils showed a continual increase, whereas naive B cells, CD8^+^ T cells, Tregs, and resting NK cells showed a continual decrease from DTC to PDTC to ATC (Fig. 2C). The types of immune cells in each specific pathological type (PTC, FTC, MTC, PDTC, and ATC) are presented (Fig. 2D). Finally, we also analyzed the association of radiation in PTC by comparing between PTC exposed to chernobyl radiation (ECR^+^) and sporadic PTC (ECR^−^). TFH was decreased whereas Tregs were increased in ECR^+^ PTC (Supplementary Fig. S2A).

### Validation of CD8^+^ T cells in clinical specimens

**Fig. 3:**
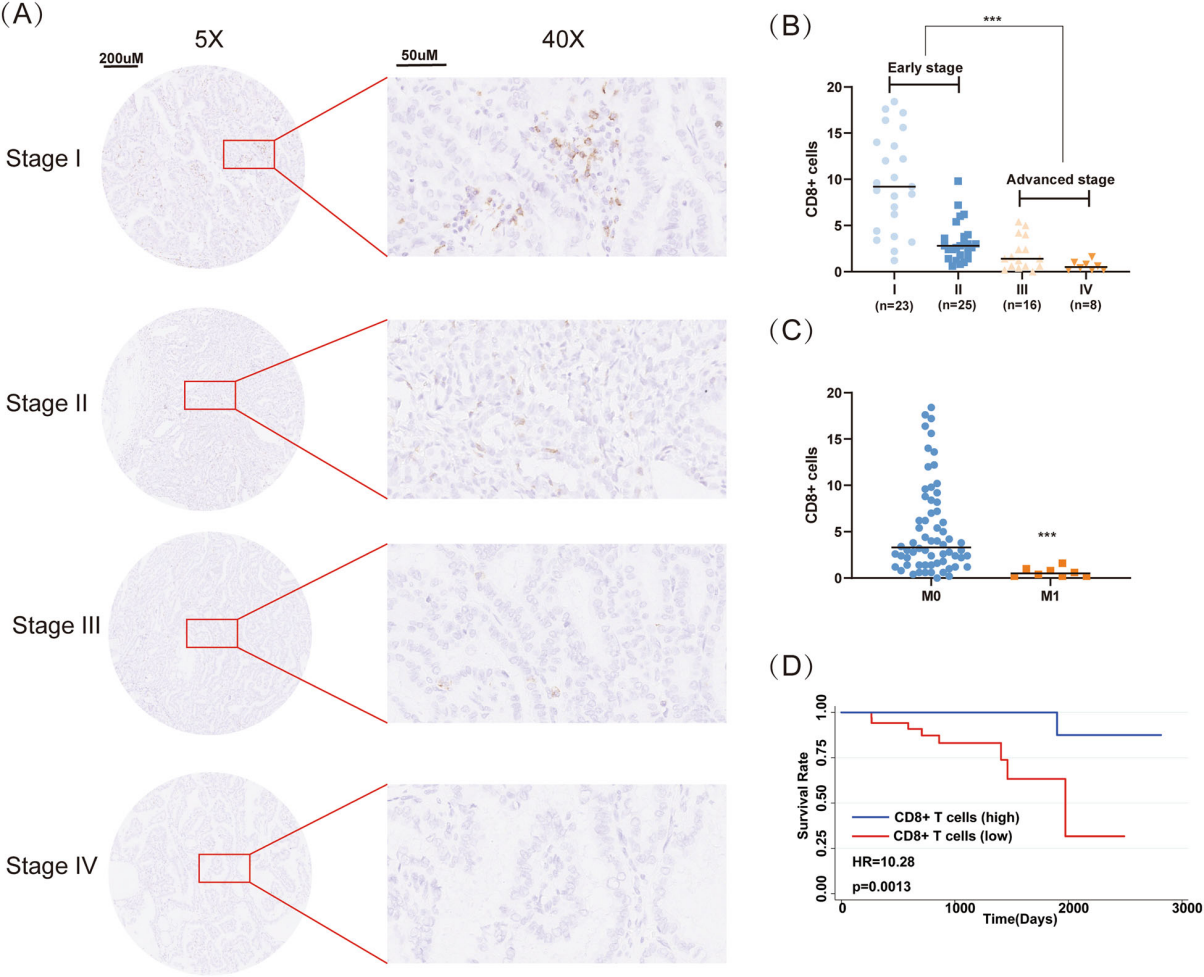
Validation of CD8^+^ T cells in clinical specimens. **A** The expression of CD8^+^ T cells detected by IHC. **B** Number of CD8^+^ T cells in different pathological stages. **C** The association of CD8^+^ T cells and distant metastasis. **D** The association of CD8^+^ T cells and OS (patients were divided based on the median of CD8^+^ T cells).

As described above, we have demonstrated CD8^+^ T cells were associated with the survival and progression of thyroid cancer patients based on the TCGA database. To further verify our findings, 72 cases of PTC specimens were collected and performed with IHC (Fig. 3A). As expected, CD8^+^ T cells were decreased in advanced stages compared with early stage (Fig. 3B). Similarly, CD8^+^ T cells were also decreased in patients with distant metastasis (Fig. 3C). Further survival analysis also tallied with the TCGA database. The low proportion of CD8^+^ T cells was associated with poor OS (Fig. 3D).

### Immune checkpoints in thyroid cancer

**Fig. 4:**
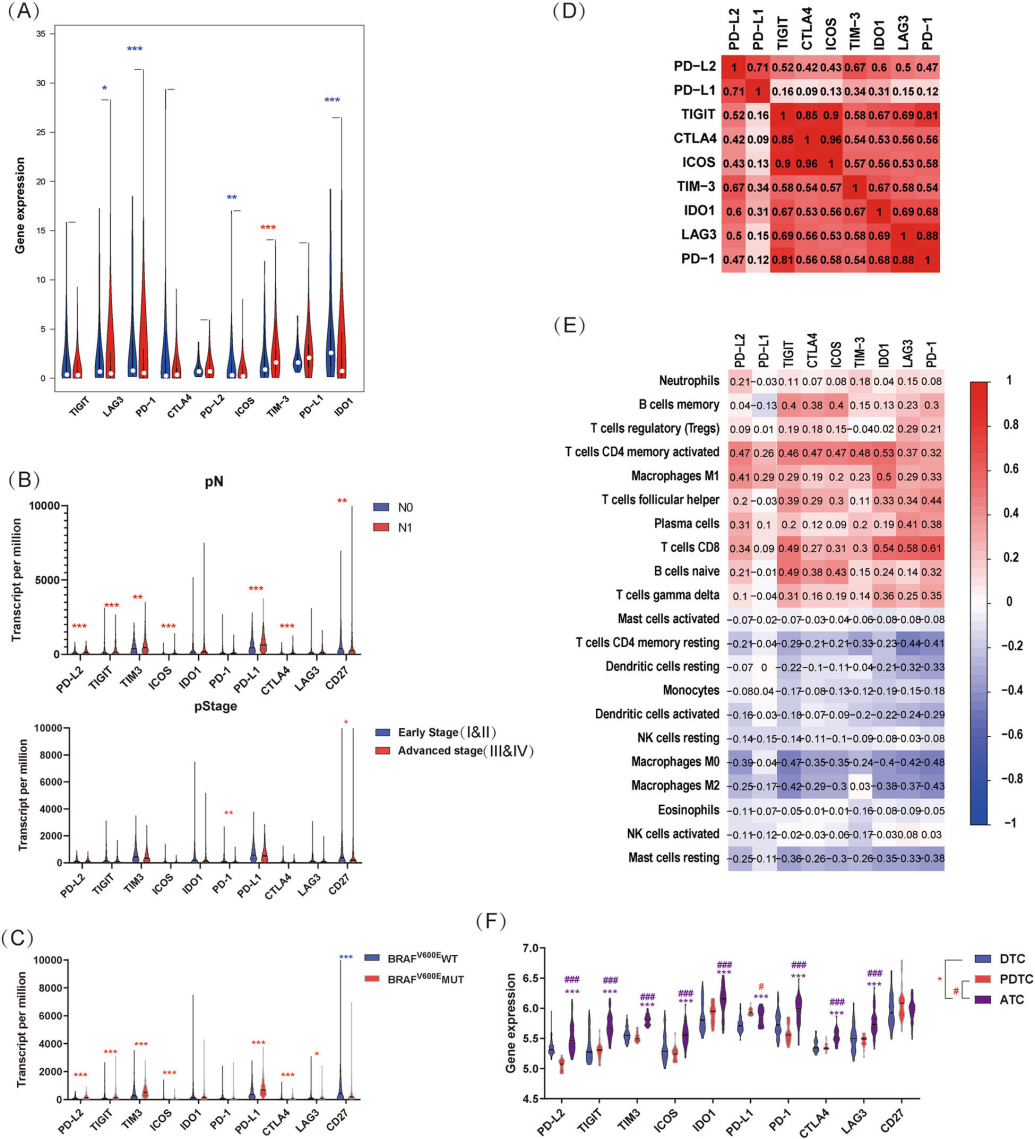
Immune checkpoints in thyroid cancer. **A** Expression of various immune checkpoints in PTC and healthy thyroid tissues. **B** Correlation of immune checkpoints and pathological stage in PTC. **C** Correlation of immune checkpoints and BRAF^V600E^ mutation in PTC. **D** Correlation matrix of immune checkpoints in PTC. **E** Correlation matrix of immune checkpoints and immune cells in PTC. **F** Correlation of immune checkpoints and different pathological types of thyroid cancer. (*P < 0.05, **P < 0.01, ***P < 0.001).

Immune checkpoints have an important role in the field of cancer immunotherapy and are a series of molecules that produce costimulatory or inhibitory signals in the immune response. We further investigated the expression of various checkpoints in PTC. We found that most checkpoint molecules, including LAG3, PD-1, ICOS, and IDO1, were significantly decreased in PTC compared with healthy thyroid tissues. Only TIM-3 was increased in PTC compared with healthy thyroid tissues (Fig. 4A). Subsequently, we investigated the expression of checkpoints in different pathological stages (Fig. 4B). Interestingly, lymph node metastasis was associated with the overexpression of various checkpoints, including PD-L2, TIGIT, TIM-3, ICOS, PD-L1, and CD27. Progression of the pathological stage was associated with overexpression of PD-1 and CD27. In terms of associations between pathological M stage and checkpoint expression, only PD-L2 was associated with distant metastasis (Supplementary Fig. S3A). We inferred that the subtle differences came from rare M1 patients in our data. In summary, the progression of the pathological stage of PTC was associated with the overexpression of various immune checkpoints. Furthermore, we investigated the association of immune checkpoints with the BRAF^V600E^ mutation. We demonstrated that most checkpoints were overexpressed in samples with the BRAF^V600E^ mutation, including PD-L2, TIGIT, TIM-3, ICOS, PD-L1, and LAG3, and only CD27 was repressed (Fig. 4C). Subsequently, we investigated the correlation of the immune checkpoints with each other, and significant co-expression was confirmed (Fig. 4D). We further investigated the correlation between immune checkpoints and immune cells. We demonstrated that neutrophils, memory B cells, Tregs, activated CD4^+^ T cells, M1 macrophages, TFH cells, plasma cells, CD8^+^ T cells, naive B cells, and γδT cells showed a positive correlation with immune checkpoints, whereas other immune cells showed a negative correlation (Fig. 4E). In addition, we investigated the expression of checkpoints in different pathological types of thyroid cancer. Interestingly, we found that most immune checkpoints in ATC were overexpressed compared with those in both DTC and PDTC, except for PD-L1 and CD27 (Fig. 4F). Specifically, there was no significant difference in the expression of CD27 in three types of thyroid cancer. In addition, PD-L1 was significantly higher in PDTC than in ATC. Finally, we investigated the association between ECR and immune checkpoints. CD27 and TIM-3 were downregulated in ECR^+^ PTC compared with ECR^−^ PTC (Supplementary Fig. S2B).

### Tertiary lymphoid structures and stromal cells in papillary thyroid cancer

**Fig. 5:**
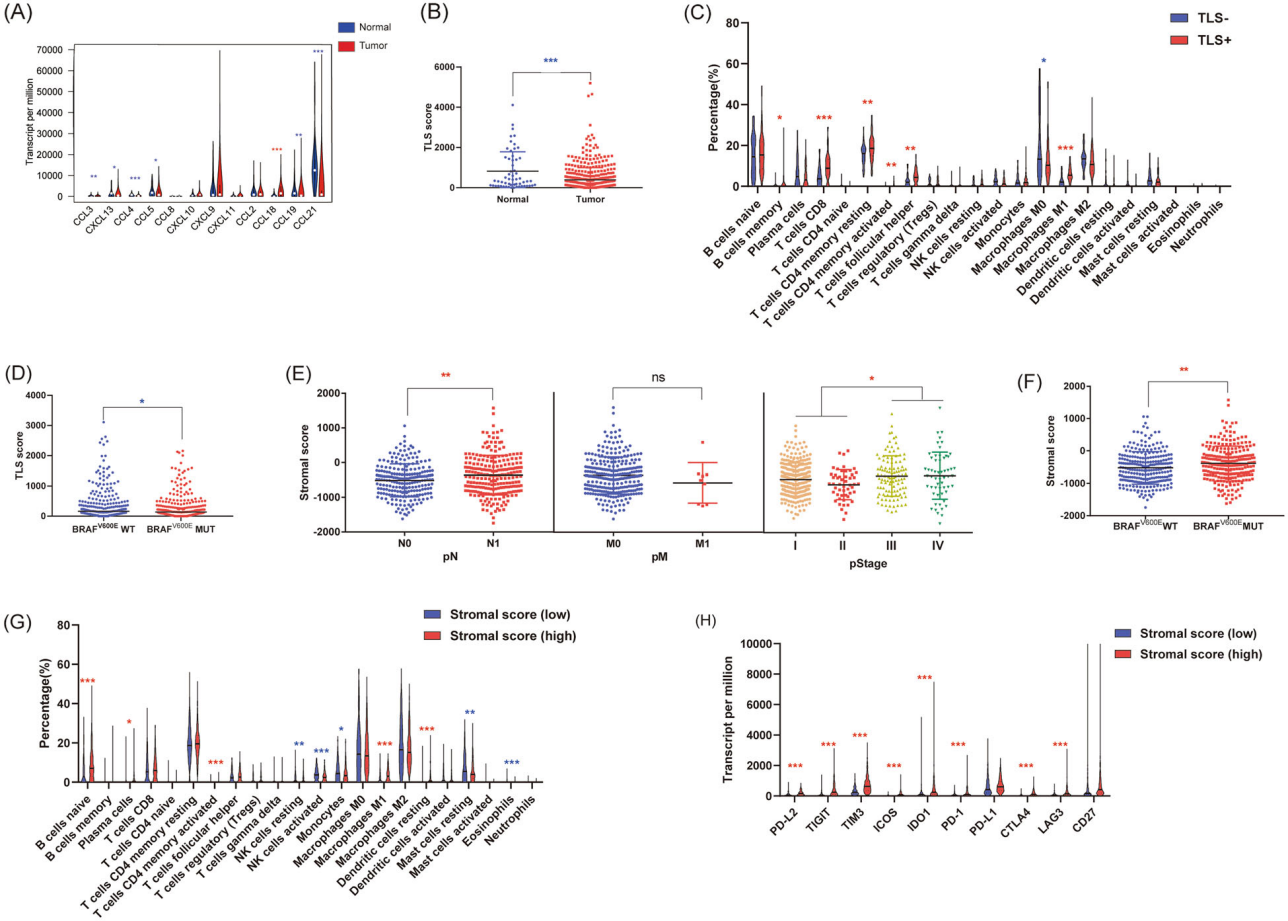
Tertiary lymphoid structures and stromal cells in papillary thyroid cancer. **A** Expression of tertiary lymphoid structure (TLS)-associated chemokines in PTC and healthy thyroid tissues. **B** TLS score of PTC and healthy thyroid tissues. **C** Correlation of TLS score and immune cells in PTC. **D** Correlation of TLS score and BRAF^V600E^ mutation in PTC. **E** Correlation of the stromal score and different pathological stages of PTC. **F** Correlation of stromal score and BRAF^V600E^ mutation in PTC. **G** Correlation of stromal score and immune cells in PTC. **H** Correlation of stromal score and immune checkpoints in PTC. (*P < 0.05, **P < 0.01, ***P < 0.001).

Infiltrating stromal and immune cells form the major fraction of normal cells in tumor tissue not only perturb the tumor signal in molecular studies but also have important roles in cancer biology. Tertiary lymphoid structures (TLSs) are considered the germinal center of immune cells in tumors. Therefore, we further investigated them in PTC. The geometric mean of a series of chemokines known to be involved in the formation of TLSs was used to assess TLSs. First, we investigated the expression of these chemokines. Most chemokines were decreased in PTC compared with healthy thyroid tissues, including CCL3, CXCL13, CCL4, CCL5, CCL19, and CCL21, and only CCL18 was increased (Fig. 5A). Consistently, the TLS score of PTC was decreased compared with that of healthy thyroid tissues (Fig. 5B). Then, we investigated the correlation of TLSs and immune cells. TLSs were associated with an increase in various immune cells in PTC, including memory B cells, CD8^+^ T cells, resting memory CD4^+^ T cells, activated memory CD4^+^ T cells, TFH cells, and M1 macrophages. Only M0 macrophages were decreased in TLS^+^ PTC compared with TLS- PTC samples (Fig. 5C). We also investigated the correlation of TLSs with immune checkpoints in PTC, and a rare difference was confirmed between TLS^−^ and TLS^+^ PTC samples (Supplementary Fig. S3B). Subsequently, we investigated the correlation of TLSs and pathological stage, and a negative correlation was confirmed (Supplementary Fig. S3C). Interestingly, we demonstrated that the BRAF^V600E^ mutation repressed TLSs in PTC (Fig. 5D). The score of stromal cells in PTC was calculated by the ESTIMATE algorithm. In contrast to TLSs, the stromal score was closely related to the pathological stage of PTC. With the advancement of both pathological N stage and pathological stage, the stromal score significantly increased (Fig. 5E). In addition, the BRAF^V600E^ mutation also indicated a higher stromal score (Fig. 5F). In immune cell analysis, a high stromal score was associated with an increase in naive B cells, plasma cells, activated memory CD4^+^ T cells, M0 macrophages, and resting DCs and a decrease in resting NK cells, activated NK cells, monocytes, resting mast cells, and eosinophils (Fig. 5G). We also determined a close correlation between the stromal score and immune checkpoints. A high stromal score indicated the overexpression of various immune checkpoints, including PD-L2, TIGIT, TIM-3, ICOS, IDO1, PD-1, CTLA4, and LAG3 (Fig. 5H).

### Cancer-associated fibroblasts and endothelial cells in thyroid cancer

**Fig. 6:**
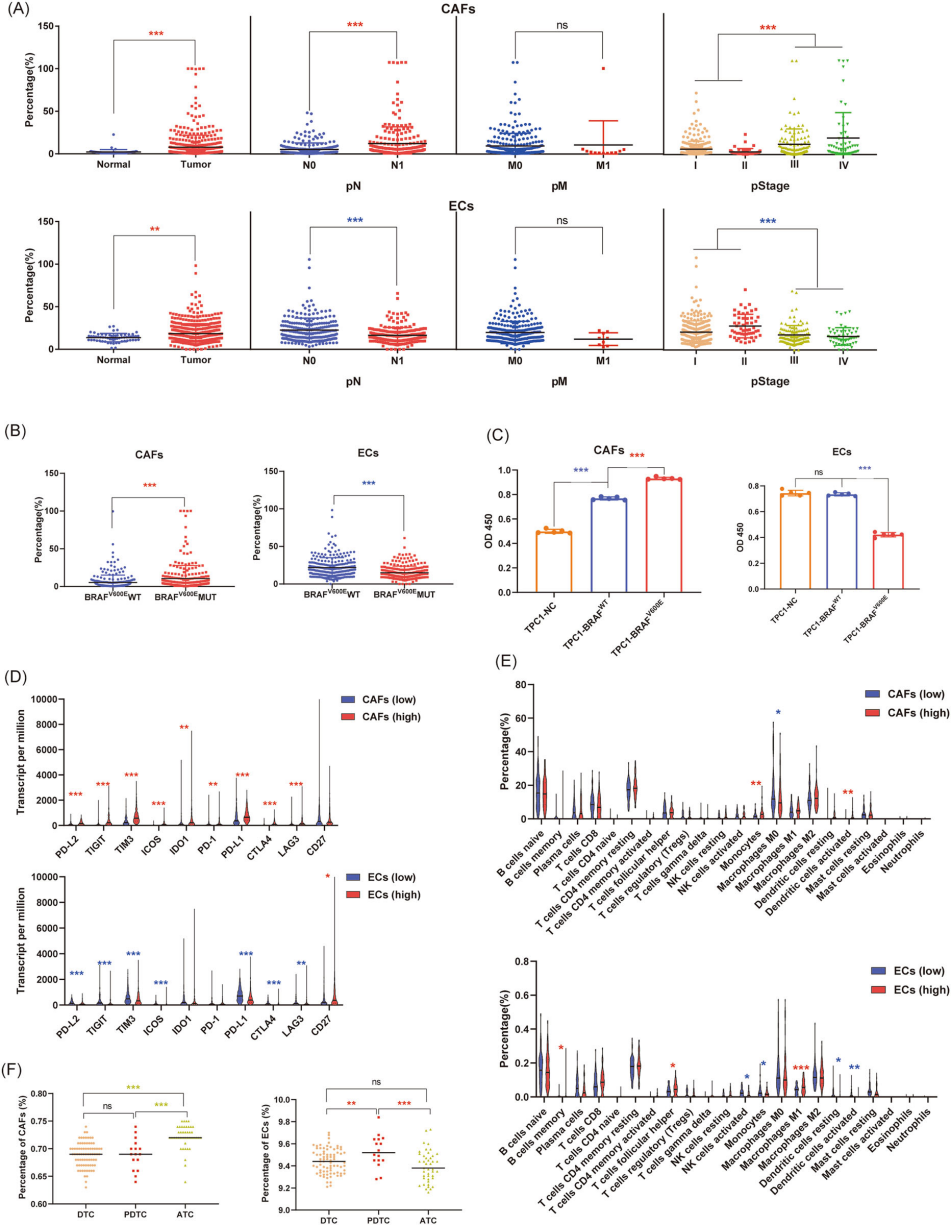
Cancer-associated fibroblasts and endothelial cells in thyroid cancer. **A** Expression of cancer-associated fibroblasts (CAFs) and endothelial cells (ECs) in different pathological stages of PTC and healthy thyroid tissues. **B** Association of the BRAF^V600E^ mutation with CAFs and ECs in PTC. **C** The proliferation of CAFs and HUVECs (ECs). Co-cultured with TPC1 transfected with wild-type (WT) BRAF and BRAF^V600E^ mutation. **D** Association of immune checkpoints with CAFs and ECs in PTC. **E** Association of immune cells with CAFs and ECs in PTC. **F** Expression of CAFs and ECs in different pathological types of thyroid cancer. (ns no significance, *P < 0.05, **P < 0.01, ***P < 0.001).

Cancer-associated fibroblasts (CAFs) and endothelial cells (ECs) are both important components of the TME and participate in the immune regulation of tumors. Therefore, we investigated the expression of CAFs and ECs in PTC. Both CAFs and ECs were increased in PTC compared with healthy thyroid tissues. The increase in CAFs but a decrease in ECs was associated with the advancement of pathological N stage and pathological stage (Fig. 6A). Similarly, the BRAF^V600E^ mutation was associated with an increase in CAFs but a decrease in ECs (Fig. 6B). Furthermore, wild-type BRAF cell line TPC1 was transfected with BRAF-wild-type (WT) plasmid and BRAF^V600E^ mutation plasmid respectively, followed by co-culturing with HUVECs or CAFs. As expect, TPC1-BRAF^V600E^ promoted the proliferation of CAFs but inhibited the proliferation of HUVECs, compared to TPC1-BRAF^wt^ (Fig. 6C). We further investigated the association of immune checkpoints with CAFs and ECs. Interestingly, CAFs were associated with the upregulation of various immune checkpoints, whereas ECs were associated with the downregulation of various immune checkpoints (Fig. 6D). Furthermore, we determined that CAFs were associated with an increase in monocytes and activated DCs and a decrease in M0 macrophages. ECs were associated with an increase in memory B cells, TFH cells, and M1 macrophages and a decrease in activated NK cells, monocytes, resting DCs, and activated DCs (Fig. 6E). Finally, we investigated the expression of CAFs and ECs in different pathological types of thyroid cancer. CAFs were mostly expressed in ATC, whereas ECs were mostly expressed in PDTC (Fig. 6F).

### **Establishment of an immune risk score model for predicting the prognosis of thyroid cancer**

**Fig. 7:**
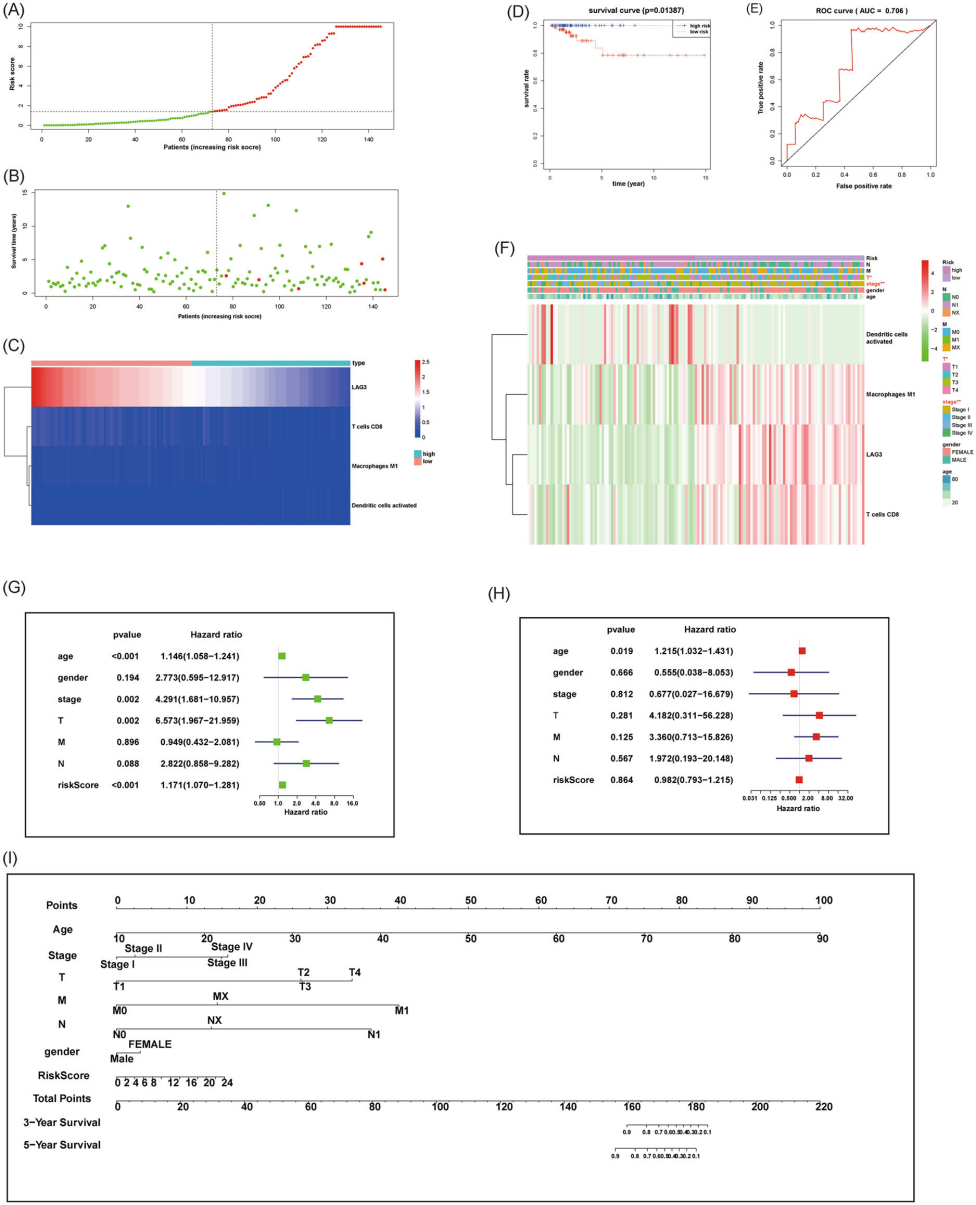
Establishment of an immune risk score for prediction of prognosis. **A** Distribution of immune risk scores in PTC patients. **B** Distribution of survival status in PTC patients. **C** Distribution of specific risk factors (LAG3, CD8^+^ T cells, M1 macrophages, and activated DCs) in the high- and low-risk groups (divided by median value). **D** Kaplan–Meier survival curves of overall survival between the high- and low-risk groups. **E** ROC curve AUC statistics for assessing the predictive capability of the immune risk score model. **F** Correlation between immune risk score and clinical characteristics. **G** Univariate Cox regression analysis for seven clinicopathological parameters affecting overall survival. **H** Multivariate Cox regression analysis for seven clinicopathological parameters affecting overall survival. **I** Established a nomogram to visualize the immune risk score model. (*P < 0.05, **P < 0.01).

To establish a scoring system for predicting the prognosis of PTC, all differentially expressed immune cells and immune checkpoints were analyzed with a single-factor Cox regression model. *P* < 0.05 was set as the screening criterion. The expression of LAG3 and the proportions of CD8^+^ T cells, M1 macrophages, and activated DCs were ultimately adopted in the multifactor Cox regression model used to construct an immune risk score (Supplementary Table S4). Based on the median value of the risk score, we divided the patients into high-risk and low-risk groups. The distribution of immune risk score, patient survival status, and expression of risk factors in TCGA-PTC patients is presented in Fig. 7A–C. Figure 7B indicated the survival times of PTC patients in the TCGA population. Figure 7C showed the expression and distribution of LAG3, CD8 T cells, macrophages M1, and activated dendritic cells between high- and low-risk groups. The Kaplan–Meier analysis suggested that patients in the high-risk group had a poor OS (Fig. 7D). The ROC curve revealed that the risk model had good sensitivity and specificity in predicting survival risk (Fig. 7E). Figure 7F showed the expression and distribution of LAG3, CD8 T cells, macrophages M1, activated dendritic cells, and risk scores in different clinical characteristics groups. In addition, there was a significant difference in the risk score between the T stage and the pathological stage (Fig. 7F). To explore whether the constructed immune risk score model was independent of other clinical-pathological parameters, we performed univariate and multivariate Cox regression analysis for age, sex, stage, TNM stage, and risk score (Fig. 7G, H). Both univariate and multivariate Cox regression analyses showed that the risk score was an independent prognostic predictor for PTC. Finally, on the basis of the coefficients derived from the multivariate Cox regression analysis, we constructed a nomogram to visualize our model (Fig. 7I). According to the nomogram, the survival of PTC patients can be predicted by age, gender, TNM stage, stage, and risk scores.

## Discussion

Immune cells in the TME have been proven to be crucial in the development of various tumors. Different types of tumors have different immune cell subpopulations. Even for the same pathological type, the subpopulation could be different among patients^26^. Therefore, it was crucial to investigate immune cell subsets to evaluate risk and tumor prognosis. Several studies have demonstrated the expression of some immune cells in thyroid cancer, including Tregs, DCs, macrophages, and T cells^15,27^. Recently, an increasing number of types and subtypes of immune cells have been reported. Therefore, previous research is not enough to reveal the whole picture of immune infiltration in thyroid cancer.

CIBERSORT, a gene expression-based deconvolution algorithm, was developed to assess the proportion of 22 types of immune cells in a mixed-cell population. Due to its excellent performance, its application in researching immune infiltration has gained importance^28^. With large sample data from the TCGA and GEO databases, we conducted a comprehensive and detailed assessment of immune infiltration in thyroid cancer. We demonstrated that thyroid cancer was locally infiltrated with various immune cell subgroups. These characteristic immune cells constituted an individualized “immune signature map” for patients with thyroid cancer and provided new ideas for subsequent specific immunotherapy.

By comparing PTC and healthy thyroid tissue, we found that naive B cells, memory B cells, CD8^+^ T cells, Tregs, γδT cells, TFH cells, M0, M1, and M2 macrophages, resting/activated DCs, and resting mast cells were differentially expressed. Most of these cell types have been reported in previous research and have different roles in the TME, such as immune evasion (Tregs, T cells, and DCs) and regulation of tumor growth and invasion (T cells and macrophages)^27,29^. However, few studies have focused on the role of naive B cells, memory B cells, TFH cells, and resting cells in PTC. These cells also take part in the development of tumors. For example, the infiltration of TFH cells was closely related to the survival of breast cancer and lung cancer^30,31^. We inferred that a similar role could also be confirmed in thyroid cancer. Subsequently, we demonstrated that infiltration of CD8^+^ T cells and neutrophils indicated better and worse OS, respectively. Similarly, CD8^+^ T cells were upregulated in DTC with a better prognosis, whereas neutrophils were upregulated in ATC with a worse prognosis. Neutrophils were reported to be associated with tumor size and invasion of thyroid cancer^32^. CD8^+^ T cells recognize and attack tumor cells expressing tumor antigens and are associated with improved disease-free survival (DFS) in DTC^33^. Therefore, our research further complements previous understanding. The BRAF^V600E^ mutation is the most common genetic alteration in thyroid cancer and promotes the invasiveness, metastasis, and recurrence of tumors^34^. The BRAF^V600E^ mutation was also associated with a decrease in CD8^+^ T cells. We also identified a series of immune cells associated with the advancement of the pathological stage. Among them, CD8^+^ T cells were associated with lymph node metastasis as well as the pathological stage. Meanwhile, we also confirmed CD8^+^ T cells were associated with the progression and survival of thyroid cancer patients in our clinical specimens. These results further proved the important role of CD8^+^ T cells in thyroid cancer. To determine CD8^+^ T cells regulatory mechanisms, we investigated CD8^+^ T cell-regulated molecules and pathways. The PI3K/Akt and PPAR pathways and SLN and TNNT1 genes were responsible for downregulated CD8^+^ T cells. Especially for TNNT1, we confirmed overexpression of TNNT1 in TPC-1 promoted the apoptosis of CD8^+^ T cells. Therefore, drugs targeting them may provide potential value in the treatment of thyroid cancer.

The expression of immune checkpoints is important for immune escape and treatment with ICIs. Several key immune checkpoints were repressed in PTC compared with healthy thyroid tissues, including LAG3, PD-1, PD-L2, and IDO1. We inferred that the higher expression of immune checkpoints in healthy thyroid tissues than in tumor tissues prevented the killing effect of immune cells on normal thyroid tissue. Interestingly, with the advancement of the pathological stage (especially N stage), most immune checkpoints were upregulated. Similarly, the BRAF^V600E^ mutation was also associated with the upregulation of most immune checkpoints. In addition, most immune checkpoints were significantly upregulated in ATC compared with their expression in DTC and PDTC. On the one hand, high expression of immune checkpoints promoted the development and invasion of thyroid cancer by the immune escape of tumor cells. On the other hand, high expression of immune checkpoints in advanced thyroid cancer also suggested increased sensitivity to ICIs. In addition, we also demonstrated a close correlation between each immune checkpoint, as well as between immune checkpoints and immune cells.

Chronic lymphocytic thyroiditis (CLT) is an autoimmune disease that can coexist with thyroid adenoma. Considering that the incidence rate of PTC combined with CLT has been increasing in recent years, we can evaluate the prognosis of thyroid cancer through the study of chronic thyroiditis and actively treat CLT to avoid carcinogenesis^35^. In the thyroid tissue of CLT patients, there were different numbers of inflammatory cells (mainly lymphocytes) with focal or scattered infiltration, even forming lymph follicles of different sizes and obvious germinal center, namely the tertiary lymphoid structures (TLS)^36^. TLS, also known as ectopic lymphoid structures (ELS), usually refers to the ectopic lymphoid structures formed in the peripheral non-lymphoid organs (liver, lung, kidney) and other chronic inflammatory sites^37,38^. It is composed of a T cell region containing mature dendritic cells (DC), B-cell follicles, high endothelial venules (HEVs), germinal centers containing follicular dendritic cells (FDC), and so on^39^. Studies are gradually revealing the mechanism of TLS in an antitumor adaptive immune response. Studies have shown that TLS density has a beneficial effect on overall survival and disease-free survival. In pancreatic cancer, lung cancer, colorectal cancer, and non-invasive breast cancer, TLS can be applied as a predictor for overall survival^38,40,41^. However, rare studies focused on thyroid cancer. Therefore, the analysis and evaluation of the immune cell population in TLS of thyroid carcinoma can provide a useful reference for immunotherapy. The recruitment and retention of lymphocytes into TLSs require various chemokines. We first investigated the expression of chemokines contributing to the formation of TLSs. Most chemokines were downregulated in thyroid cancer, including CCL3, CCL4, CCL15, CCL21, and CXCL13. Correspondingly, TLSs were decreased in thyroid cancer. In addition, various immune cells were repressed with a decrease in TLSs. This result explains the role of TLSs in promoting immune infiltration. Interestingly, the BRAF^V600E^ mutation was also associated with a decrease in TLSs. This is consistent with the poor prognosis associated with the BRAF^V600E^ mutation.

In addition to tumor cells and immune cells, there are abundant stromal cells in the TME. Stromal cells regulate immune infiltration by secreting cytokines or activating signaling pathways^42^. Through the ESTIMATE algorithm, each case of thyroid cancer was assigned a stromal score (no cases of healthy thyroid tissues received a stromal score, as the concept of stromal cells was not applicable in normal tissue). Interestingly, we demonstrated that an increase in stromal score was associated with the advancement of the pathological stage as well as the BRAF^V600E^ mutation. Stromal cells seem to indicate a poor prognosis in thyroid cancer. In addition, an increase in stromal cells also promoted the expression of various immune checkpoints. This means that stromal cells promoted immune escape of tumor cells as well as increased sensitivity to ICIs. CAFs and ECs are the two most important stromal cells in tumors. We further investigated their roles in immune infiltration, and the two showed completely opposite characteristics. However, both CAFs and ECs were increased in thyroid cancer compared with normal tissues. Upregulated CAFs but downregulated ECs were associated with the advancement of pathological stage and the BRAF^V600E^ mutation. In addition, CAFs promoted the expression of various immune checkpoints, whereas ECs repressed them. Similarly, CAFs promoted the infiltration of activated DCs, whereas ECs repressed it. DCs were reported to promote immune escape in PTC^27^. These results indicate that CAFs promoted immune escape and the development of thyroid cancer, whereas ECs showed opposite characteristics. Immunotherapy targeting these two cell types may provide new ideas.

At present, effective biomarkers for predicting the prognosis of thyroid cancer are still lacking^43^. Based on our research, we constructed an immune risk score model to predict the prognosis of thyroid cancer based on a Cox regression model. The model showed the promising value in predicting both survival risk and pathological stage. Finally, we also established a nomogram to visualize the model.

In conclusion, our study has revealed an immune infiltration map in thyroid cancer. We revealed the complex relationships between immune cells, immune checkpoints, tumor stromal cells, TLC, prognosis, survival, BRAF^V600E^ mutation, pathological stage, pathological types, and so on. Our research may provide new opinions regarding the mechanisms of thyroid cancer development and immune therapy.

## Supplementary information

Table S1

Table S2

Table S3

Table S4

Figure S1

Figure S2&3

## Data Availability

The data sets used and analyzed during the current study are available from the corresponding author on reasonable request.
