# Supplementary material for: A new risk factor indicator for papillary thyroid cancer based on immune infiltration
Source: Cell Death Dis. 2021 Jan 6;12(1):51. doi: 10.1038/s41419-020-03294-z (PMC7791058; doi:10.1038/s41419-020-03294-z)
Supplement: Supplementary file 1 — Table S1 [file 41419_2020_3294_MOESM1_ESM.docx]

Table 1 Clinical characteristics of patients

| Factors |  | Number |
| --- | --- | --- |
| Age | <=45 | 231 |
|  | >45 | 279 |
| Gender | Female | 371 |
|  | Male | 139 |
| Stage | Stage I | 288 |
|  | Stage II | 52 |
|  | Stage III | 113 |
|  | Stage IV | 57 |
| T stage | T1 | 143 |
|  | T2 | 167 |
|  | T3 | 175 |
|  | T4 | 23 |
|  | TX | 2 |
| M stage | M0 | 287 |
|  | M1 | 9 |
|  | MX | 214 |
| N stage | N0 | 229 |
|  | N1 | 231 |
|  | NX | 50 |
| BRAF^V600E^ | Wide Type | 262 |
|  | Mutant | 248 |
| TX, MX, NX: indeterminate pathological stage. | | |
