# Supplementary material for: A new risk factor indicator for papillary thyroid cancer based on immune infiltration
Source: Cell Death Dis. 2021 Jan 6;12(1):51. doi: 10.1038/s41419-020-03294-z (PMC7791058; doi:10.1038/s41419-020-03294-z)
Supplement: Supplementary file 2 — Table S2 [file 41419_2020_3294_MOESM2_ESM.docx]

Table 2 Clinical characteristics of patients

| Factors |  | Number |
| --- | --- | --- |
| Age | <=45 | 32 |
|  | >45 | 40 |
| Gender | Female | 49 |
|  | Male | 23 |
| Stage | Stage I | 23 |
|  | Stage II | 25 |
|  | Stage III | 16 |
|  | Stage IV | 8 |
| T stage | T1 | 14 |
|  | T2 | 23 |
|  | T3 | 24 |
|  | T4 | 11 |
| M stage | M0 | 64 |
|  | M1 | 8 |
| N stage | N0 | 50 |
|  | N1 | 22 |
