# Supplementary material for: A new risk factor indicator for papillary thyroid cancer based on immune infiltration
Source: Cell Death Dis. 2021 Jan 6;12(1):51. doi: 10.1038/s41419-020-03294-z (PMC7791058; doi:10.1038/s41419-020-03294-z)
Supplement: Supplementary file 3 — Table S3 [file 41419_2020_3294_MOESM3_ESM.docx]

Table S3 P-values for Figure 2A

| Types | pStage |  | pT |  | pN |  | pM |
| --- | --- | --- | --- | --- | --- | --- | --- |
| B cells naive | 0.727034 |  | 0.376969 |  | 0.003524 |  | 0.310206 |
| B cells memory | 0.407881 |  | 0.51994 |  | 0.601129 |  | 0.640911 |
| Plasma cells | 0.00109 |  | 0.005625 |  | 0.224301 |  | 0.278045 |
| T cells CD8 | 0.000405 |  | 0.159855 |  | 0.000615 |  | 0.287333 |
| T cells CD4 memory resting | 0.093558 |  | 0.395757 |  | 0.013412 |  | 0.569951 |
| T cells CD4 memory activated | 0.416873 |  | 0.624892 |  | 0.853808 |  | 0.695569 |
| T cells follicular helper | 0.025551 |  | 0.181627 |  | 0.93958 |  | 0.428985 |
| T cells regulatory (Tregs) | 0.55656 |  | 0.857964 |  | 0.531942 |  | 0.596771 |
| T cells gamma delta | 0.088042 |  | 0.298888 |  | 0.466774 |  | 0.662315 |
| NK cells resting | 0.962793 |  | 0.700882 |  | 0.631184 |  | 0.900917 |
| NK cells activated | 0.292963 |  | 0.933943 |  | 0.029794 |  | 0.861218 |
| Monocytes | 0.0046 |  | 6.31E-05 |  | 0.796447 |  | 0.754366 |
| Macrophages M0 | 0.151359 |  | 0.098611 |  | 0.119527 |  | 0.108384 |
| Macrophages M1 | 0.002852 |  | 0.223907 |  | 0.485741 |  | 0.118641 |
| Macrophages M2 | 0.208141 |  | 0.353785 |  | 0.17283 |  | 0.68761 |
| Dendritic cells resting | 0.020917 |  | 0.126404 |  | 0.248206 |  | 0.26439 |
| Dendritic cells activated | 0.018164 |  | 0.019687 |  | 0.136346 |  | 0.03875 |
| Mast cells resting | 0.013359 |  | 0.318994 |  | 0.28577 |  | 0.754241 |
| Mast cells activated | 0.031119 |  | 0.095526 |  | 0.269466 |  | 0.976148 |
| Eosinophils | 0.134975 |  | 0.027825 |  | 0.713497 |  | 0.088182 |
| Neutrophils | 0.433136 |  | 0.779564 |  | 0.606772 |  | 0.03144 |
