# Supplementary material for: A new risk factor indicator for papillary thyroid cancer based on immune infiltration
Source: Cell Death Dis. 2021 Jan 6;12(1):51. doi: 10.1038/s41419-020-03294-z (PMC7791058; doi:10.1038/s41419-020-03294-z)
Supplement: Supplementary file 4 — Table S4 [file 41419_2020_3294_MOESM4_ESM.docx]

Table 3 The univariate Cox regression analysis for differentially expressed immune cells and immune checkpoints in papillary thyroid cancer patients

| Factors | P-value | HR | 95%CI | | |
| --- | --- | --- | --- | --- | --- |
| Activated DCs | 0.013* | 1.84E+11 | 262.0332 | 1.30E+20 | |
| CD8+ T cells | 0.035* | 5.60E-11 | 1.69E-20 | 0.186143 | |
| M1 Macrophage | 0.045* | 2.47E-14 | 1.23E-27 | 0.497307 | |
| M2 Macrophage | 0.111 | 115940.6 | 0.069535 | 1.93E+11 | |
| M0 Macrophage | 0.136 | 39.85403 | 0.312116 | 5088.947 | |
| Memory B cells | 0.142 | 248071.6 | 0.015276 | 4.03E+12 | |
| Follicular helper T cells | 0.316 | 2.48E-06 | 2.73E-17 | 224921.6 | |
| Gama delta T cells | 0.725 | 3475.657 | 6.97E-17 | 1.73E+23 | |
| Resting DCs | 0.866 | 2.99773 | 8.77E-06 | 1024787 | |
| Naive B cells | 0.868 | 3.983771 | 3.13E-07 | 50715067 | |
| LAG3 | 0.038* | 0.202582 | 0.044861 | 0.914809 | |
| PD-1 | 0.113 | 0.544021 | 0.256164 | 1.155351 | |
| IDO1 | 0.150 | 0.688839 | 0.414913 | 1.14361 | |
| TIM-3 | 0.408 | 1.152033 | 0.824011 | 1.610634 | |
| ICOS | 0.658 | 1.228097 | 0.494044 | 3.052809 | |
| HR: hazard ratio; CI: confidence interval; *P＜0.05 | | | | |  |
