# Supplementary figures and images for: A new risk factor indicator for papillary thyroid cancer based on immune infiltration
Source: Cell Death Dis. 2021 Jan 6;12(1):51. doi: 10.1038/s41419-020-03294-z (PMC7791058; doi:10.1038/s41419-020-03294-z)

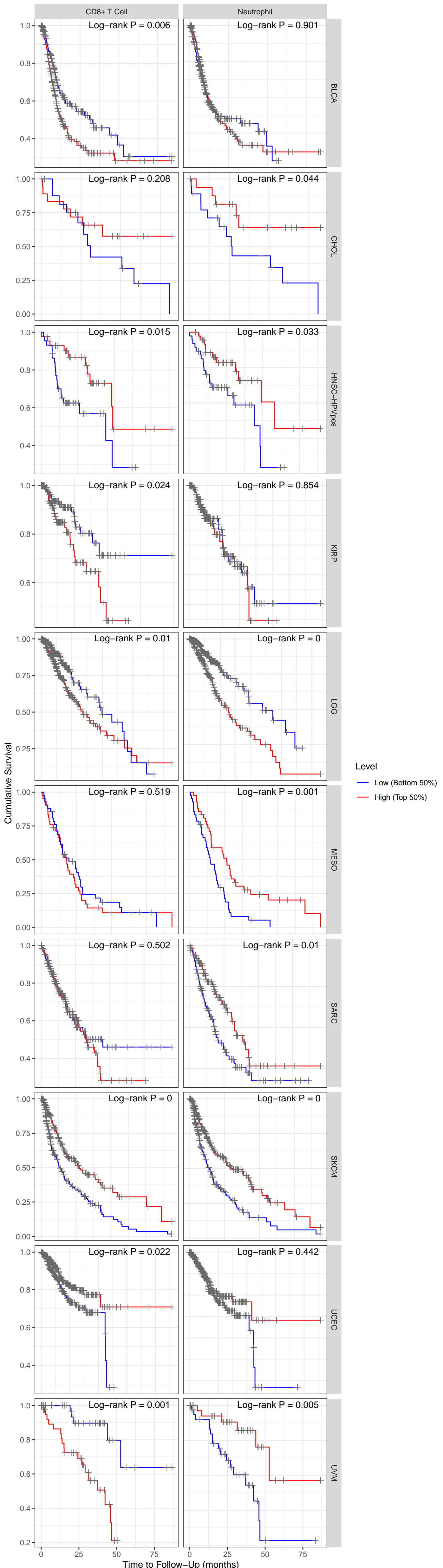

Supplement: Supplementary file 5 — Figure S1 [file 41419_2020_3294_MOESM5_ESM.pdf]
