# Supplementary material for: A new risk factor indicator for papillary thyroid cancer based on immune infiltration
Source: Cell Death Dis. 2021 Jan 6;12(1):51. doi: 10.1038/s41419-020-03294-z (PMC7791058; doi:10.1038/s41419-020-03294-z)
Supplement: Supplementary file 6 — Figure S2&3 [file 41419_2020_3294_MOESM6_ESM.docx]

Supplementary Figure 1. The association of Overall Survival with CD8+ T cells and neutrophils analyzed by TIMER website tool. (BLCA: bladder urothelial carcinoma; CHOL: cholangiocarcinoma; HNSC-HPVpos: head and neck squamous cell carcinoma with HPV; KIRP: kidney renal papillary cell carcinoma; LGG: lowed grade glioma; MESO: mesothelioma; SARC: sarcoma; SKCM: skin cutaneous melanoma; UCEC: uterine corpus endometrial carcinoma; UVM: uveal melanoma).


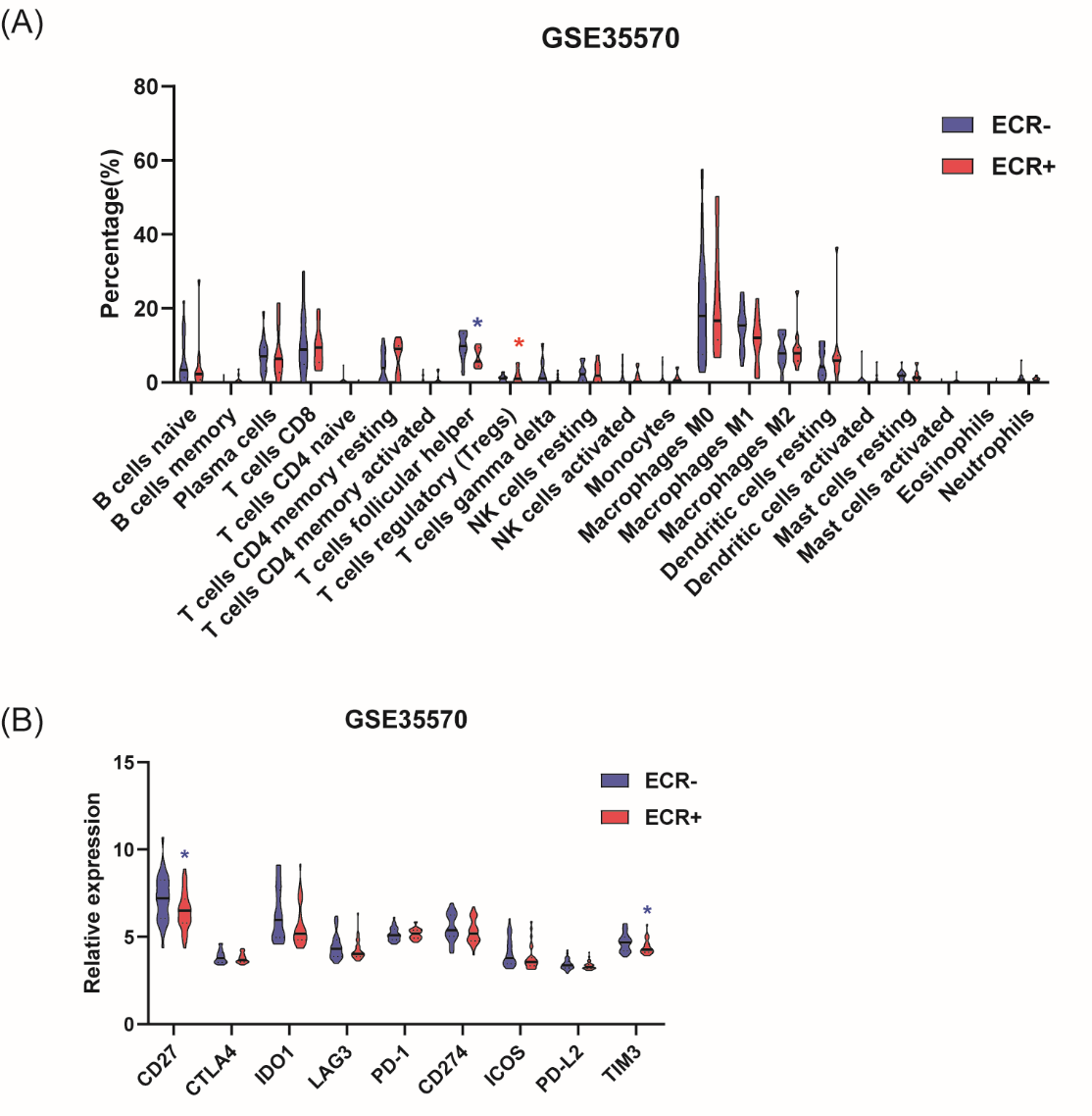


Supplementary Figure 2. A. Compare of immune cells between PTC exposed to Chernobyl Radiation (ECR+) and sporadic PTC (ECR-). B. Compare of immune checkpoints between PTC exposed to Chernobyl Radiation (ECR+) and sporadic PTC (ECR-). (**P<0.05).


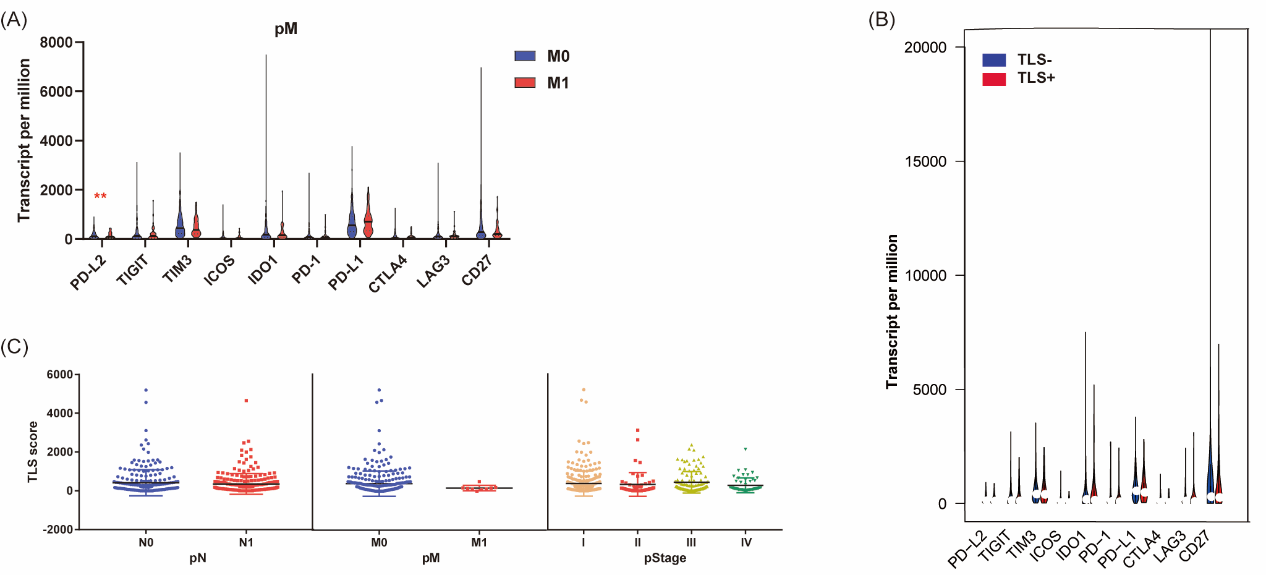


Supplementary Figure 3. A. Association of pathological M stage and expression of immune checkpoints. (**P<0.01). B. Expression of immune checkpoints in TLS- and TSL+ PTC. (All showed no significance.). C. Correlation of TLS score and pathological stage in PTC. (All showed no significance.)
